# Effect of early enteral nutrition support for the management of acute severe pancreatitis: A protocol of systematic review

**DOI:** 10.1097/MD.0000000000021569

**Published:** 2020-08-07

**Authors:** Yong-bo Guo, Yan Liu, Jing Ma, Ying Cai, Xiao-ming Jiang, Hong Zhang

**Affiliations:** a Department of Critical Care Medicine, The Affiliated Hongqi Hospital of Mudanjiang Medical University; b Department of Critical Care Medicine, First Affiliated Hospital of Mudanjiang Medical University, Mudanjiang; c Department of Critical Care Medicine, Beijing Mentougou District Hospital, Beijing, China.

**Keywords:** acute severe pancreatitis, enteral nutrition support, effect

## Abstract

**Background:**

This study aims to assess the effect of early enteral nutrition support (EENS) for the management of acute severe pancreatitis (ASP).

**Methods:**

This study will search Cochrane Library, PUBMED, EMBASE, Cumulative Index to Nursing and Allied Health Literature, Allied and Complementary Medicine Database, CNKI, and WANGFANG from their inception to the present without language limitations. In addition, this study will also search clinical trial registry and reference lists of included trials. Eligible comparators will be standard care, medications, and any other interventions. Two authors will independently scan all citations, titles/abstracts, and full-text studies. The study methodological quality will be appraised using Cochrane risk of bias tool. If it is possible, we will pool out data and perform meta-analysis. Strength of evidence for each main outcome will be evaluated using the Grading of Recommendations Assessment, Development, and Evaluation.

**Results:**

This study will summarize the most recent evidence to assess the effect of EENS for the management of ASP.

**Conclusion:**

The findings of this study will help to determine whether EENS is effective for patients with ASP.

**Study registration:**

INPLASY202070009.

## 1 Introduction

Acute severe pancreatitis (ASP) is one of the most common inflammatory gastrointestinal diseases occurring in the pancreas with high mortality,^[1–3]^ which is caused by bile stones or excessive alcohol drinking.^[4–7]^ It has been estimated that its global incidence varies from 5 to 30 cases/100,000 population annually.^[8,9]^ It is often associated with single or multiple organ dysfunction and infectious complications.^[10]^ Thus, it needs urgent intensive care and management.

Early enteral nutrition support (EENS) has been reported to reduce septic complications, surgical procedures, and decrease length of hospital stay for patients with ASP.^[11–21]^ However, there are inconsistent conclusions of EENS for the management of ASP.^[11–21]^ In addition, no systematic review has been conducted focusing this issue. Thus, this systematic review will investigate the effect of EENS for the management of patients with ASP.

## 2 Methods

### 2.1 Study registration

This study protocol was registered at INPLASY202070009. Its reports follow the guidelines of Preferred Reporting Items for Systematic Reviews and Meta-Analysis Protocol statement.^[22,23]^

### 2.2 Eligibility criteria

#### 2.2.1 Study types

The present study will include potential randomized controlled trials (RCTs) focusing on the effect of EENS for the management of ASP. We will exclude experimental study, case report, case series, non-clinical trials, uncontrolled trials, and non-RCTs.

#### 2.2.2 Intervention types

Interventional group: Patients who received EENS will be included.

Control group: Patients who received any management will be considered as a comparator. However, we will exclude comparators involved any forms of EENS.

#### 2.2.3 Participant types

Patients with confirmed diagnosis of ASP will be included, irrespective educational background, race, gender, age, and duration of ASP.

#### 2.2.4 Outcome measurement types

Primary outcomes include levels of serum endotoxin, lactulose/mannitol ratio of urine, and tumor necrosis factor.

Secondary outcomes are C-reactive proteins, white blood cell, interleukin-6, mortality rate, infection rate, and length of hospital stay.

### 2.3 Search strategy

Electronic databases will be searched from inception onwards to the present in Cochrane Library, PUBMED, EMBASE, Cumulative Index to Nursing and Allied Health Literature, Allied and Complementary Medicine Database, CNKI, and WANGFANG. We will not limit language and publication status. We will provide search strategy temple of Cochrane Library in Table 1. Similar search strategies will be adapted for other electronic databases. In addition, we will perform relevant documents or reviews, website of clinical trial registers, and reference lists of eligible studies.

**Table 1:**
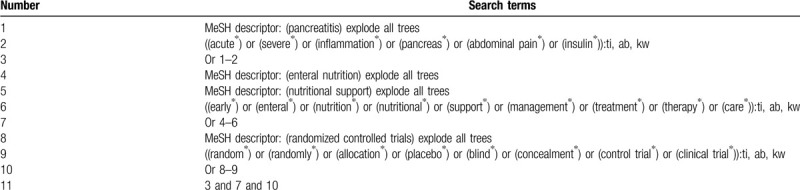
Search strategy of Cochrane Library.

### 2.4 Study selection

Study selection will be performed based on the eligibility criteria, and its process consists of two stages. At the first step, all duplicates and irrelevant literatures will be removed after screening titles/abstracts of all searched records. At the second step, full manuscripts of all potential articles will be read against all inclusion criteria. Any divergences will be solved by discussion and a consensus conclusion will be drawn. The process will be tracked in a flowchart.

### 2.5 Data extraction process

Two independent authors will extract data using a pre-specified data collection form. It consists of publication details (such as title, year of publication, et al), patient information (such as gender, age, et al), specifics of study methods, treatments and controls (such as types of delivery, dosage, et al), outcome indicators, safety, and other essential information. Any conflicts will be cleared up by a third author. If essential data is unclear or missing, the original authors are contacted to request it.

### 2.6 Risk of bias assessment

All eligible studies will be critically appraised by two independent authors using Cochrane risk of bias tool. It includes 7 aspects, and each item is divided into three levels: low, unclear or high risk of bias. In case of disagreements, the results will be discussed and settled down by a third author.

### 2.7 Treatment effect measurement

Treatment effect of continuous data will be estimated as standardized mean difference and 95% confidence intervals (CIs), and that of dichotomous data will be calculated as risk ratio and 95% CIs.

### 2.8 Heterogeneity assessment

The heterogeneity across eligible trials will be assessed using *I*^*2*^ test. It is defined as follows: *I*^2^ ≤50% suggests minor heterogeneity, and we will use a fixed-effect model, while *I*^2^ > 50% means obvious heterogeneity, and we will utilize a random-effect model.

### 2.9 Data synthesis

We will utilize RevMan 5.3 software to perform data analysis. Whenever minor heterogeneity is identified across included studies, we will carry out quantitative synthesis of outcome results and will perform meta-analysis if two or more trials which report a similar outcome. Whenever remarkable heterogeneity is examined, we will perform subgroup analysis to explore its possible causes. If we can still test obvious heterogeneity after subgroup analysis, data will not be pooled, and meta-analysis will be not conducted. If necessary, we will report study results using narrative description.

### 2.10 Subgroup analysis

Subgroup analysis will be performed according to the different details of treatments and controls, different study quality and outcome indicators.

### 2.11 Sensitivity analysis

Sensitivity analysis will be carried out to test robustness of synthesized results by eliminating low quality studies.

### 2.12 Reporting bias

We will check reporting bias using Funnel plot and Egger's regression test if over 10 eligible trials are included.

### 2.13 Ethics and dissemination

We will not analyze individual patient data, thus no ethics approval is needed. The results of this study will be published on a peer-reviewed journal.

## 3 Discussion

Previous studies have hypothesized that EENS has been utilized for the management of patients with ASP.^[11–21]^ However, all of them have been conceptual. Considering an increasing number of clinical studies on investigating the effect of EENS for ASP, this systematic review aims to conduct a systematic synthesis to inform the effect of EENS for patients with ASP. We wish to summarize the most recent high-quality evidence of EENS for ASP. Its results are expected to inform patients, clinicians and researchers of future studies.

## Author contributions

**Conceptualization:** Yong-bo Guo, Yan Liu, Ying Cai, Xiao-ming Jiang, Hong Zhang.

**Data curation:** Yong-bo Guo, Jing Ma, Ying Cai, Xiao-ming Jiang.

**Formal analysis:** Yong-bo Guo, Yan Liu, Hong Zhang.

**Investigation:** Hong Zhang.

**Methodology:** Yong-bo Guo, Ying Cai, Xiao-ming Jiang.

**Project administration:** Hong Zhang.

**Resources:** Yong-bo Guo, Yan Liu, Ying Cai, Xiao-ming Jiang.

**Software:** Yong-bo Guo, Yan Liu, Jing Ma, Ying Cai, Xiao-ming Jiang.

**Supervision:** Hong Zhang.

**Validation:** Yong-bo Guo, Yan Liu, Ying Cai, Hong Zhang.

**Visualization:** Yong-bo Guo, Yan Liu, Xiao-ming Jiang, Hong Zhang.

**Writing – original draft:** Yong-bo Guo, Jing Ma, Ying Cai, Hong Zhang.

**Writing – review & editing:** Yong-bo Guo, Yan Liu, Jing Ma, Xiao-ming Jiang, Hong Zhang.
